# Interactions between Obsessional Symptoms and Interpersonal Ambivalences in Psychodynamic Therapy: An Empirical Case Study

**DOI:** 10.3389/fpsyg.2017.00960

**Published:** 2017-06-09

**Authors:** Shana Cornelis, Mattias Desmet, Kimberly L. H. D. Van Nieuwenhove, Reitske Meganck, Jochem Willemsen, Ruth Inslegers, Jasper Feyaerts

**Affiliations:** ^1^Department of Psychoanalysis and Clinical Counseling, Ghent UniversityGhent, Belgium; ^2^Centre for Psychoanalytic Studies, University of EssexColchester, United Kingdom

**Keywords:** obsessional symptoms, interpersonal characteristics, psychodynamic psychotherapy, empirical single case study, theory-building case study, ambivalence, symptom specificity

## Abstract

The classical symptom specificity hypothesis (Blatt, 1974) particularly associates obsessional symptoms to interpersonal behavior directed at autonomy and separation from others. Cross-sectional group research, however, has yielded inconsistent findings on this predicted association, and a previous empirical case study (Cornelis et al., in press; see Chapter 2) documented obsessional pathology to be rooted in profound ambivalences between autonomous and dependent interpersonal dynamics. Therefore, in the present empirical case study, concrete operationalizations of the classical symptom specificity hypothesis are contrasted to alternative hypotheses based on the observed complexities in Chapter 2. Dynamic associations between obsessional symptoms and interpersonal functioning is further explored, aiming at further contribution to theory building (i.e., through suggestions for potential hypothesis-refinement; Stiles, 2009). Similar to the first empirical case study (Chapter 1), Consensual Qualitative Research for Case studies is used to quantitatively and qualitatively describe the longitudinal, clinical interplay between obsessional symptoms and interpersonal dynamics throughout the process of supportive-expressive psychodynamic therapy. In line with findings from Chapter 1, findings reveal close associations between obsessions and interpersonal dynamics, and therapist interventions focusing on interpersonal conflicts are documented as related to interpersonal and symptomatic alterations. Observations predominantly accord to the ambivalence-hypothesis rather than to the classical symptom specificity hypothesis. Yet, meaningful differences are observed in concrete manifestations of interpersonal ambivalences within significant relationships. Findings are again discussed in light of conceptual and methodological considerations; and limitations and future research indications are addressed.

## Introduction

The centrality of interpersonal dynamics to the emergence and maintenance of symptoms has always been stressed in psychoanalytic theory. From the beginning, Freud (1978/1915c) situated the “cause” of neurotic psychopathology at the level of the libidinal organization. This was theorized to determine character formation, the accompanying relational characteristics, and the phenomenology of psychopathological symptoms Freud (1978/1908b). Since Freud, a pivotal aim of psychoanalytic research has been to identify and describe specific interpersonal dimensions, and their associations with particular symptom patterns.

In this context, the symptom specificity hypothesis of Blatt (1974, pp. 155–157) discerns two major interpersonal styles, which are differentially associated with distinctive types of neurotic symptoms. On the one hand, the *autonomous* style is hypothesized to be associated with obsessive-compulsive symptoms (e.g., obsessional ideas, compulsions, pathological doubt, inhibition), which are viewed to be distorted attempts to install a sense of self-definition and separation from others. The *dependent* style, on the other, is related to bodily symptoms (e.g., conversion reactions) and phobias, seen as exaggerated attempts toward closeness to significant others.

In order for theories to be clinically useful (i.e., grant the opportunity to inform every day clinical practice) and provide coherent, precise accounts of the phenomena under study, they need to be empirically tested in research endeavors that enable to indicate areas where theories potentially need to grow (e.g., Stiles, 2009). Over the past decades, Blatt's symptom specificity hypothesis has been put to the test in several cross-sectional group studies, which failed to yield converging results (for a review, see Desmet, 2007). It has been remarked that this lack of convergence might be due to conceptual and methodological shortcomings of the studies addressing symptom specificity. Recently, Cornelis et al. (in press) raised several of these issues related to nomothetic research designs.

*Conceptually*, it was argued that the concrete operationalizations of the classical symptom specificity hypothesis that were tested in cited studies, possibly yielded an underestimation of the complexity of associations (see also Desmet, 2013). Importantly, Blatt's theory primarily intended to define a complex, clinical interplay between symptomatic and interpersonal characteristics over time.

Hence, *methodologically*, pertinent investigation into these dynamics requires longitudinal, clinical data, in which co-variations between both levels can be analyzed over time or throughout the course of a treatment process. However, up until now, all studies that tested symptom specificity:

- Were cross-sectional in nature (i.e., relying on measurements of symptoms and interpersonal characteristics on one single time point) and, thus, described static associations;
- Focused on modal, invariant patterns in (large) groups of participants, thereby providing rule-based, abstract knowledge in which both intra-individual variability and (potentially relevant) contextual factors were disregarded as noise;
- Applied solely quantitative, patient-reported assessment of symptoms and interpersonal characteristics (i.e., by means of self-report measures, which are known to be subject to a variety of biases; e.g., Schwarz, 1999; Desmet, 2007).

Cornelis et al. (in press) concluded, therefore, that rather than focusing on additional statistical testing of the classical symptom specificity hypothesis in nomothetic research designs, there might first be a need to refine it on some points. Empirical case research specifically allows for hypothesis-refinement and theory building (e.g., Stiles, 2009) in a clinically useful manner (e.g., Edwards et al., 2004). Rigorously conducted case studies bear the ability to extend de-contextualized, rule-based knowledge on established theories, by incorporating (intra- and extra-therapeutic) contextual influences into thick descriptions of naturally unfolding processes and interactions over time. It has been argued that useful clinical theories need to account for both *patterns* amongst the complexity of psychotherapeutic processes, as well as specific *variations* and the applicability of group-based findings to the *idiographic* contexts of every day clinical practice, i.e., in which dynamic and multiple factors operate in ongoing processes, and in which consumers of research prove to be particularly interested (e.g., Flyvbjerg, 2006; Stiles, 2009; McLeod, 2013).

In an effort to meet the raised shortcomings and to detect areas where potential refinement of the classical hypothesis is necessary, Cornelis et al. (in press) put forward a research methodology (discussed below) that was specifically tailored for addressing dynamic associations between symptoms and interpersonal dynamics throughout longitudinal therapy processes. The present study applies this methodology to test symptom specificity in an empirical case study of a patient with obsessional complaints. The patient was treated in a real-world clinical practice by means of supportive-expressive psychodynamic therapy (Luborsky, 1984).

Concretely, the aim of the paper is 2-fold:

1. To test concrete operationalizations of the classical symptom specificity hypothesis (as presented below)
2. To thoroughly investigate the dynamic unfolding of associations between the patient's symptomatic and interpersonal functioning throughout therapy.

The additional discovery-oriented nature of the design thus scopes for the detection of distinctive, unexpected findings that could indicate where the classical hypothesis potentially needs to grow. In this way, we address recommendations of both earlier research on symptom specificity to make use of longitudinal designs (e.g., Pilkonis, 1988) in mental health clinical settings (e.g., Huprich et al., 2013; Werbart and Forsström, 2014), as broader claims in psychotherapy research to direct future research endeavors toward the increased use of idiographic research (e.g., Barlow and Nock, 2009; Iwakabe and Gazzola, 2009; Stiles, 2009; Dattilio et al., 2010; Hill, 2012; McLeod, 2013; Vanheule, 2014).

The applied methodology (Cornelis et al., in press) compiles a combination of Consensual Qualitative Research for Case studies (CQR-c; Jackson et al., 2011), which serves as the overarching data-analytic approach, and the “Core Conflictual Relationship Theme” method (CCRT; Luborsky and Crits-Cristoph, 1998), as a means of systematizing empirical investigation of interpersonal behavior.

CQR-c has specifically been developed to assess complex clinical material in a rich and nuanced fashion. By addressing the data through different angles in a team of researchers, a broad dialog amongst competing perspectives is explicitly installed throughout multiple team meetings, until all team members agree on the best representation of the data (Hill et al., 1997). This “triangulation” process is claimed to result in a more meaningful understanding of the studied phenomena (Dattilio et al., 2010) and to significantly contribute to the “credibility” (i.e., the qualitative parallel of validity; Morrow, 2005) of the results.

CCRT methodology, as a widely used method in psychotherapy research, is based on Luborksy (1962) theory that subjects' relational exchanges are underpinned by a typical “core conflict.” This conflict is comprised of three major components (Luborsky and Crits-Cristoph, 1998): the wishes, needs or intentions with which a subject enters relational exchanges (“Wish,” W); the subject's appraisal of how the other person responds to these wishes (“Response of Other,” RO); and his/her own responses to these ROs (“Response of Self,” RS).

As symptoms are theorized to be deeply rooted in the subject's core conflict, Luborksy (1962, 1984) further claimed that psychotherapeutic endeavors aiming at transforming this core conflict will bring about symptomatic changes, which has previously been evidenced by Grenyer and Luborsky (1996), Luborsky and Crits-Cristoph (1998), and Slonim et al. (2011). Hence, in accordance with the supportive-expressive therapy (Luborsky, 1984) under study, the applied CCRT-method provides conformity between the treatment as conducted by the therapist, and the researchers' method of analyzing the narrative data extracted from this treatment.

Next, with the aim to illuminate different aspects of (the wide spectrum of possible changes in) the studied variables (e.g., Hill et al., 2013), extensive multiple method and multiple source data sets were analyzed. Symptomatic and interpersonal functioning, and their according associations, were assessed regularly throughout treatment and follow-up, in both a quantitative and qualitative fashion, from perspectives of patient, therapist, and researchers. Symptoms and associated mental distress were additionally mapped via saliva cortisol concentrations (i.e., hormonal biomarkers of distress) and health care costs (i.e., information on all mental and physical health related expenses and job absenteeism; see Method Section).

Recently, this combination of CQR-c and CCRT methodology has been applied for symptom specificity research in a previous empirical case study of a patient with obsessional complaints (Cornelis et al., in press). Importantly, this study shed light on complexities that were not captured by the classical symptom specificity hypothesis, and thus resulted in a suggested refinement. Close associations were observed

- Between the patient's symptomatic and interpersonal functioning,
- Between therapist interventions focusing on interpersonal conflicts and interpersonal and symptomatic transformations.

Yet, instead of the predicted predominance of *autonomous* interpersonal behavior, obsessional symptoms were observed to be rooted in profound *ambivalences* between autonomy and dependency. More specifically, recent separating attempts to break out of long-established dependent interpersonal issues, meaningfully determined the patient's obsessions. These ambivalences were observed both *within* significant relationships (i.e., in alternating loving and vindictive relational exchanges within each relationship), as *between* significant relations (i.e., alternatively preferring one relationship above the other), and thus suggested more complex interpersonal dynamics than originally assumed by the classical symptom specificity hypothesis. Yet, the suggested complexity proved in accordance with the hypothesis' broader theoretical underpinnings. Both classical (e.g., Freud, Lacan) and contemporary (e.g., Blatt, Luborsky) psychodynamic theories document separating tendencies in close association with feelings of ambivalence, i.e., out of fear of losing the love of significant others (e.g., Verhaeghe, 2001).

Aiming to contribute to a rich, nuanced understanding of symptom specificity, the present “theory-building” case study (Stiles, 2009) will further explore clinical complexity of associations between obsessional symptoms and specific interpersonal dynamics. For that purpose, concrete operationalizations of the classical symptom specificity hypothesis (Blatt, 1974, pp. 155–157) are contrasted to alternative hypotheses based on cited findings of Cornelis et al. (in press).

Operationalizing interpersonal characteristics by means of the CCRT-method, the classical symptom specificity hypothesis leads up to the following **predictions** with respect to symptomatic-interpersonal associations in the patient under study:

*H1:* Before therapy (during the intake phase) we expect the obsessional symptoms to be accompanied by an autonomous interpersonal style, expressed in an exaggerated emphasis on self-definition and separation from others.
*H1a:* Quantitatively, we expect the patient will show an autonomous sub-profile on the Inventory of Interpersonal Problems (IIP-32), rather than a dependent sub-profile (see Desmet et al., 2013).
*H1b:* Qualitatively, we expect the following CCRT-components (Luborsky and Crits-Cristoph, 1998) to underpin the patient's relational exchanges: Wishes (with which he enters exchanges) = independence, self-control, self-assertion, being acknowledged and respected, achieving; Responses of Other (i.e., his appraisal of how the other person responds to these wishes) = critical, controlling, opposing, not respectful; Responses of Self (i.e., his own subsequent responses) = anxiety, self-doubt/uncertainty, guilt, feelings of failure, (struggles with) aggression, vengeful fantasies.
*H2:* Throughout the therapeutic process, we expect that the supportive-expressive therapy will reduce the exaggerated strivings toward autonomy and that, as a consequence, obsessive-compulsive symptoms will diminish.
*H2a:* Quantitatively, we expect that scores on the IIP-autonomy profile will decrease progressively throughout therapy and that the decreasing IIP-scores will be correlated with decreasing scores on symptoms and general distress.
*H2b:* Qualitatively, we expect that changes in the autonomous CCRT's throughout therapy (particularly in the RO- and RS-components, e.g., Crits-Christoph and Luborsky, 1990; Grenyer and Luborsky, 1996) will be accompanied by changes in the obsessive-compulsive symptoms.

Then, based on the observed complexities reported in Cornelis et al. (in press), the following **alternative predictions** are advanced:

*H3:* Obsessional symptoms to be rooted in *ambivalences* between a marked autonomous *and* dependent interpersonal style, expressed in profound emphasis on self-definition and separation from others, as a means of escaping interpersonal struggles with dependency.
*H3a:* Quantitatively, we expect the patient will report more interpersonal problems with dependency compared to autonomy, depicted in a higher dependent than autonomous sub-profile on the Inventory of Interpersonal Problems (IIP-32, see Desmet et al., 2013).
*H3b:* Qualitatively, we expect, in addition to the predicted autonomous components (see *H1b*), that the patient's relational exchanges will be underpinned by persistent dependent W's to be loved by and close to significant others, RO's of rejection and distance, and RS's aimed at avoiding losing others' love.
*H4:* The supportive-expressive therapy will reduce the interpersonal struggles with dependency and support the strivings toward autonomy, and obsessional symptoms will subsequently diminish.
*H4a:* Quantitatively, we expect that scores on both IIP sub-profiles will decrease throughout therapy, and that decreasing IIP-scores will be correlated with decreasing scores on symptoms and general distress.
*H4b:* Qualitatively, we expect that changes in CCRT's throughout therapy (particularly in the RO- and RS-components, e.g., Crits-Christoph and Luborsky, 1990; Grenyer and Luborsky, 1996; Cornelis et al., in press) will be accompanied by changes in the obsessional symptoms.

## Method

### Participants

The patient was a Caucasian man, 26-year old at the start of therapy, who was referred for treatment by his general practitioner, due to daily occurring anxiety attacks that centered on the theme of suffering or dying from heart failure. Patient was a university graduate and worked as a salesman at a wholesale business. At intake, he met *Diagnostic and Statistical Manual of Mental Disorders* (DSM-IV-TR; American Psychiatric Association, 2000) criteria of Obsessive-Compulsive Disorder (axis I; no personality disorder was diagnosed on axis II). Patient provided written informed consent (approved by the University Ethics Committee) to participate in the study and to publish the individual case materials. All possibly identifying information has been changed to protect confidentiality.

The therapist was a Caucasian, 36-year old man who held a PhD in clinical psychology. Besides his job as assistant professor at the university, he worked in a private group practice. He received a three-year postgraduate training in Freudian-Lacanian psychoanalytic psychotherapy. At the start of therapy, he had 6 years of clinical experience.

The research team that carried out the data analyses was composed of a female assistant professor, two postdoctoral researchers (one male, one female) and two female doctoral students. All research team members were trained or following training in psychoanalytic psychotherapy from a Freudian-Lacanian orientation, were Caucasian and ranged in age between 25 and 35 years.

### Therapy

**Figure 1:**
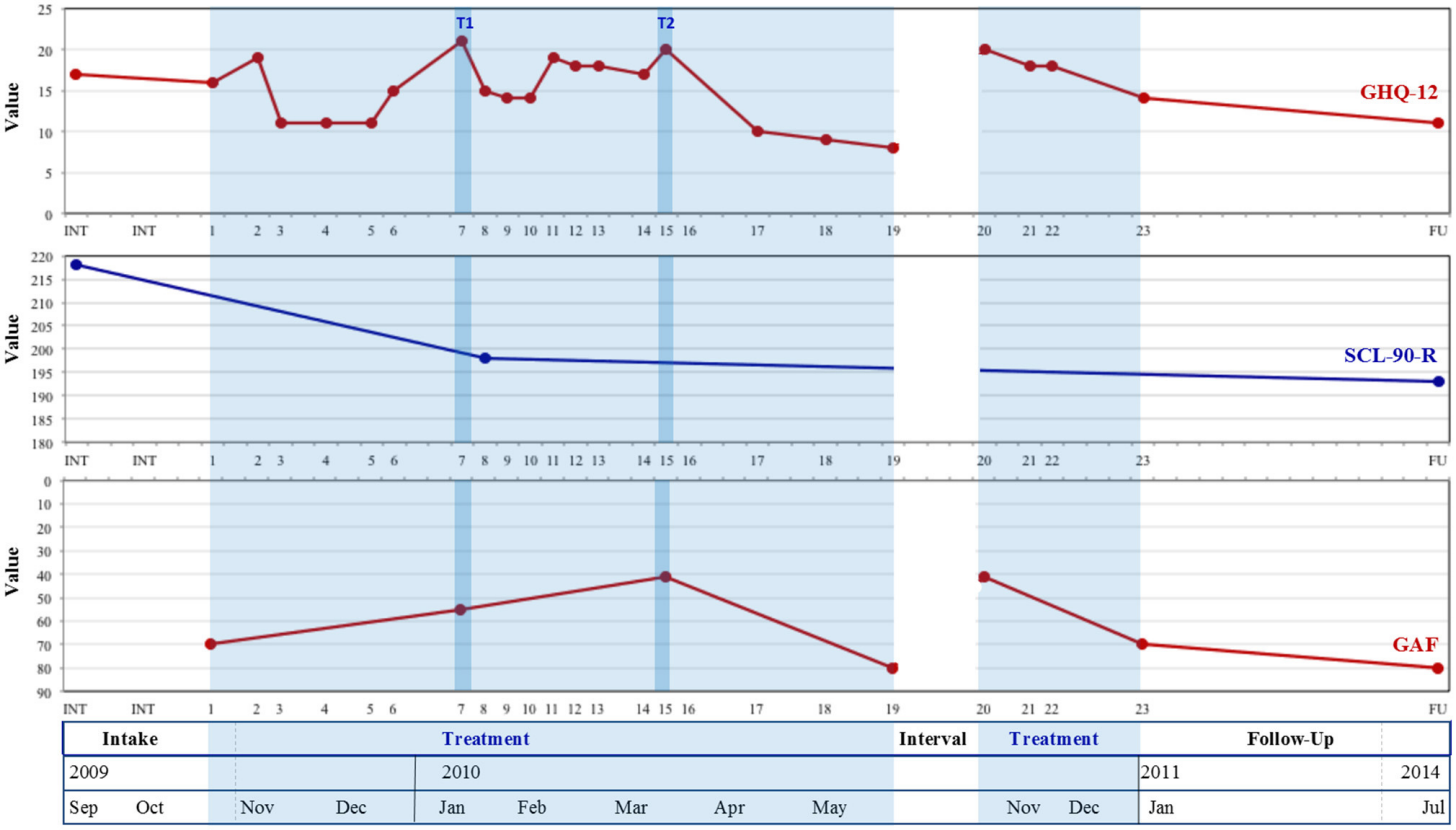
Evolutions in patient-reported (GHQ-12, SCL-90) and researcher-rated (GAF) well-being from intake to follow-up. GHQ-12, General Health Questionnaire-12; SCL-90-R, Symptom Checklist-90-Revised; GAF, Global Assessment of Functioning; T1, Tipping point 1; T2, Tipping Point 2; Treatment was interrupted between sessions 19 and 20.

In total, the patient received 23 (30- to 60-min) sessions of supportive-expressive psychoanalytic psychotherapy (Luborsky, 1984) over 15 months, conducted in the therapist's private practice, without interference of the research team. Actual frequency of the sessions varied between once a week and once every month, with an average frequency of one session every 2 weeks (see Figure 1 for a time line). Step 3 of the Results section provides specific examples of supportive and expressive techniques framed within the treatment process.

### Measures

#### Symptoms and general well-being

##### The symptom checklist-90–revised

The symptom checklist-90–revised (SCL-90-R; Derogatis et al., 1973) is a 90-item self-report questionnaire assessing general psychological and physical functioning with good psychometric qualities (Derogatis, 1994). Items are scored on a 5-point Likert scale.

##### The global assessment of functioning

The global assessment of functioning (GAF; American Psychiatric Association, 2000) scale is a widely used clinician- or researcher rated measure of psychiatric symptom severity and functioning on a psychological, social and occupational level. The scale can be used to track clinical progress of individual patients in global terms. The overall GAF scale scores range from 0 to 100 and are divided into 10 deciles of functioning.

##### The general health questionnaire-12

The general health questionnaire-12 (GHQ-12; Goldberg, 1972; Koeter and Ormel, 1991) is a 12-item self-report questionnaire used to assess general psychological distress. Items are scored using a 4-point Likert scale. The GHQ's validity and reliability was demonstrated by Koeter and Ormel (1991), and by Vanheule and Bogaerts (2005) for the Dutch version.

##### Saliva stress hormone levels

Concentrations of cortisol (μg/dl) were measured in saliva samples by means of mass-spectrometry, following the standard practice in salivary hormone research (e.g., Kirschbaum et al., 1992). Cortisol is considered a biomarker of an activated stress response. It plays a key role in numerous models that link (chronic) stressors to psychiatric as well as medical disease (Miller et al., 2007).

##### Health care costs

Health care costs were retrieved via the patient's health insurance fund, spanning from 2 years before intake until follow-up, i.e., 18 months after treatment termination. Costs include medication use (psychotropic and other), medical consultations (general practitioner and experts) and job absenteeism.

##### The semi-structured change interview

The semi-structured change interview (SCI; Elliott, 1999; Elliott et al., 2001) is an in-depth qualitative outcome interview, used to assess the way the patient experienced the therapeutic process, the changes that occurred during therapy, and the processes that might have brought about these changes.

#### Interpersonal functioning

##### The inventory of interpersonal problems-32.

The inventory of interpersonal problems-32 (IIP-32; Horowitz et al., 2000) is a 32-item self-report questionnaire with eight subscales reflecting different interpersonal problems. Items are scored on a 5-point Likert scale. Psychometric properties of the Dutch version were positively evaluated by Vanheule et al. (2006). Desmet et al. (2008) developed a scoring system for an anaclitic/hysterical and an introjective/obsessional IIP profile.

##### The core conflictual relationship theme (CCRT) method

The core conflictual relationship theme (CCRT) method (Luborsky and Crits-Cristoph, 1998) is a qualitative, systematized and reliable measure of the central relationship patterns that pervade self-other interactions (Wilczek et al., 2000). This method started from the narratives the patient spontaneously recounted during therapy about his relational exchanges. Within these narratives, two researchers selected Relationship Episodes (RE's), defined as relatively discrete episodes in which the patient explicitly speaks about concrete exchanges with others and/or himself. RE's are decomposed in three major components (see Section Introduction): (1) “Wishes” (W), (2) “Responses of Other” (RO), and (3) “Responses of Self” (RS). The most typical W's, RO's, and RS's constitute the final CCRT-formulation. In this paper, CCRT-coding was part of Step 2 of the data-analytic procedure described below.

### Procedure

Data collection happened according to the following procedure: (1) all therapy sessions were audiotaped by the therapist, transcribed verbatim by a postgraduate research assistant and checked for accuracy by two members of the research team; (2) after every session, the patient completed the IIP-32 and GHQ-12 questionnaires in the treatment practice in the therapist's presence; (3) after every session, the therapist made a brief session report in which he summarized significant dynamics at the level of symptomatology and interpersonal functioning; (4) after the first session, after every eighth session, and at follow-up (18 months after treatment termination), the patient completed a more extensive set of questionnaires at home, including IIP-32, GHQ-12, SCL-90-R, and BDI-II; (6) at follow up, SCI was administered and health care cost information was retrieved by a research team member.

### Data analysis

In order to enhance “credibility” (Morrow, 2005) and “trustworthiness” (e.g., Elliott et al., 1999; Hill, 2012) of the study, the Consensual Qualitative Research for Case Studies (CQR-c) method (Jackson et al., 2011), was used an overarching data-analytic approach. Data-analysis happened in three main steps: a quantitative and qualitative outline of evolutions in patient's symptomatology (Step 1), in interpersonal functioning (Step 2), and in associations between symptoms and interpersonal dynamics, embedded within a broader description of the therapeutic process (Step 3).

**Figure 2:**
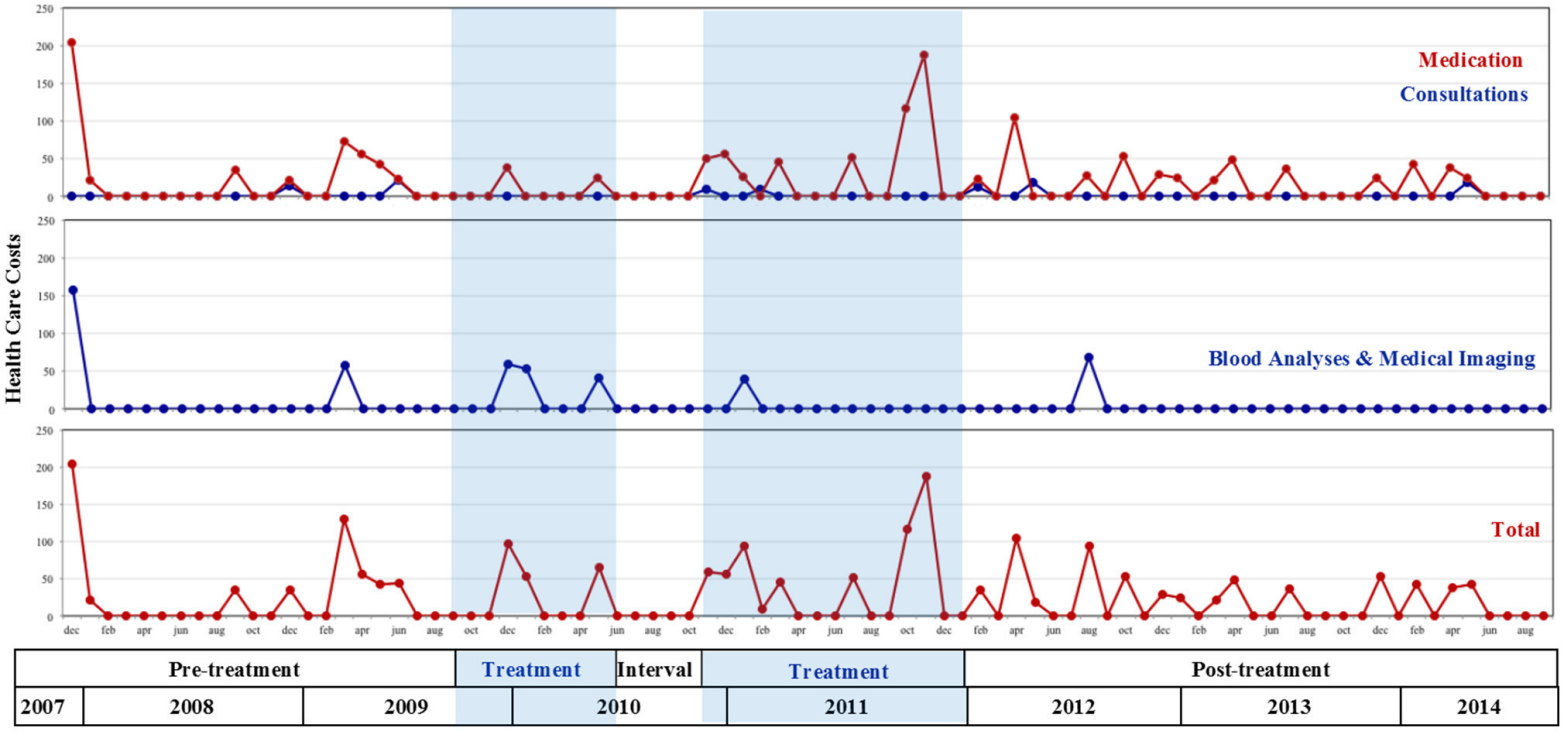
Evolutions in patient's health care costs (euro) from two years before onset of treatment until follow-up; Treatment was interrupted between sessions 19 and 20.

In Step 1, one member of the research team (referred to below as “researcher 1”) constructed graphs on quantitative evolutions in all outcome measures of symptoms and general well-being (see Figures 1, 2). To assess significance of change, Reliable Change Indices (RCI; identical to the RCI formula of Jacobson and Truax (1991), but with one-tailed 95% confidence intervals; see Brown et al., 2015) and severity adjusted effect sizes (SAES; Brown et al., 2015) were calculated by means of the ACORN Toolkit (specifically designed to help clinicians and researchers calculate change related statistics for a variety of outcome measures, used in a variety of clinical settings; see Brown et al., 2015). Next, two research team members (i.e., “researchers 1 and 2”) familiarized themselves with the narrative data by attentively listening to audiotapes and reading the transcripts. Both members were equally informed of relevant patient demographic information and treatment characteristics (as described in “Method” above; see also Hill, 2012), but researcher 2 was blind to the quantitative graphs. Both researchers separately identified all events where the patient explicitly referred to his obsessional symptom, and marked symptomatic evolutions throughout therapy with respect to intensity, content or form. Through subsequent discussion on the most profound symptomatic changes, consensus was reached on identification of the main “tipping points” (i.e., specific moments in the chronicle of events that turn out to be crucial for further development; Tarrow, 2004). In case of divergence, discussions were installed in which the researchers questioned each other on their ideas, so that every opinion was fully expressed and understood (see also Schielke et al., 2009; Jackson et al., 2011), until both members agreed on the best representation of the data (Hill et al., 1997). Next, sessions in which the selected tipping points occurred, were visually marked on the quantitative graphs. Finally, researcher 1 provided a concise qualitative description of the discussed symptomatic evolutions (see Results, Step 1), which was reviewed by a third team member who had knowledge of the raw narrative data, and was consequently refined.

**Figure 3:**
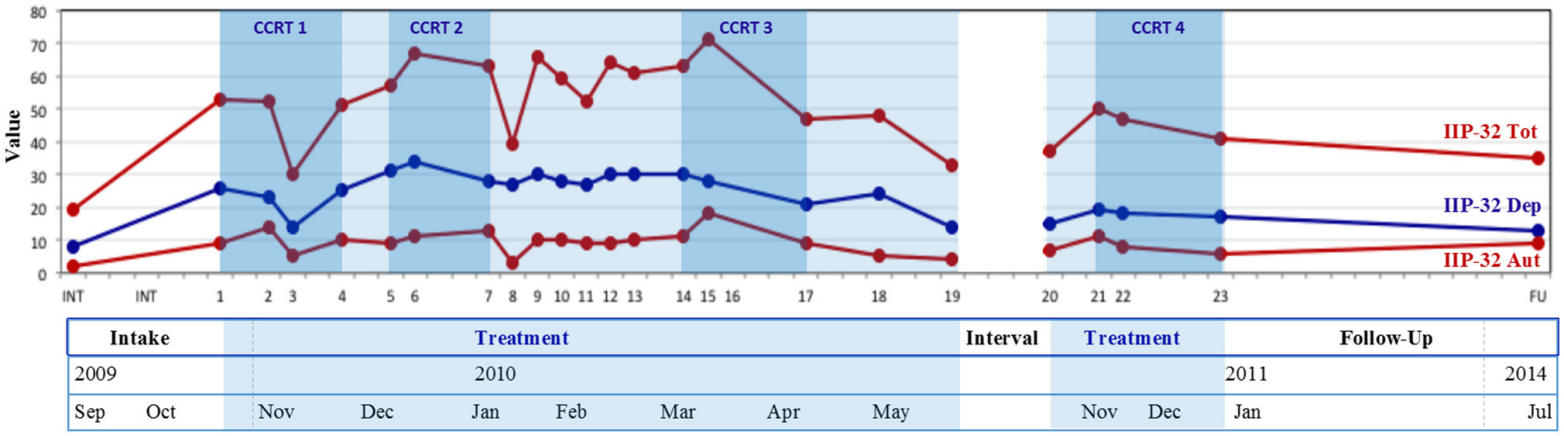
Evolutions in patient-reported interpersonal problems from intake to follow-up. IIP-32 total, Inventory of Interpersonal Problems-32 total scores; IIP-32 Dep, Inventory of Interpersonal Problems-32 subscores dependency; IIP-32 Aut, Inventory of Interpersonal Problems-32 subscores autonomy; CCRT1, Conflictual Relationship Theme codings of therapy sessions 1–4; CCRT2, Conflictual Relationship Theme codings of Tipping point 1 sessions; CCRT3, Conflictual Relationship Theme codings of Tipping point 2 sessions; CCRT4, Conflictual Relationship Theme codings of last three sessions. Treatment was interrupted between sessions 19 and 20.

In Step 2, researcher 1 constructed similar graphs on evolutions throughout therapy in interpersonal characteristics (see Figure 3), including IIP-32 total scores, and dependent and autonomous IIP-32 sub-profiles (see Vanheule et al., 2006). RCI and SAES were computed using the ACORN Toolkit (Brown et al., 2015) to assess significance of change. Next, for (1) the first therapy sessions, (2) the “tipping point”-sessions selected in Step 1, and (3) the last sessions, CCRT analyses were conducted. Researchers 1 and 2 acted as CCRT-raters. In a first phase, both attentively read the transcripts of the identified sessions again, and individually selected all RE's that were suitable for CCRT coding (i.e., RE's that contained W's, RO's, and RS's). Subsequently, judges gathered to select by consensus the 10 most informative RE's. When a (“tipping point”) session yielded less than 10 informative RE's, additional RE's were selected from the session preceding and following this (“tipping point”) session. In a second phase, selected RE's were written down in a separate document and coded using the standardized coding system (Standard Category List, Edition 2; Luborsky and Crits-Cristoph, 1998, p. 26), i.e., the one best-fitting category for each W, RO and RS is chosen from the approximately 30 categories on the standard CCRT scoring-sheet. To help ensure a richer representation of the data, and in line with Hill et al. (2011), judges distinguished between RE's describing interactions with*specific people* and RE's describing interactions with people *in general*. Further in line with Hill et al. (2011), judges distinguished between W's, RO's and RS's occurring in *all* RE's with the interacting person (General, G), in *at least half* of RE's (Typical, T), and in *less than half, but at least two* RE's (Variant, V). Judges strived toward consensus on identified RE's (Step 2, phase 1) and CCRT-codes of identified RE's (Step 2, phase 2). During consensus meetings, they alternately read aloud their individual ratings and subsequently compared them to those of the other. When agreement existed, judges proceeded to the following RE (phase 1) or CCRT-code (phase 2). In case of divergence, researchers engaged in extensive discussions as described in Step 1. Throughout this process, judges gradually refined their initial ratings by integrating valuable contributions of the other, until consensus codes were reached (see Hill, 2012). Judges' proportions of agreement (RE's:0.74, W's:0.71, RO's:0.72, RS's:0.80) indicated acceptable correspondence for initial ratings. Finally, consensus CCRT-codes were represented by researcher 1 in organized tables (see Tables 1, **4**), and checked for accuracy and comprehensiveness by researcher 2.

**Table 1:** Patient's wishes (W), responses of other (RO), and responses of self (RS) in sessions 1–4.

**Target of interaction**	**#**	**W**	**RO**	**RS**
Anna	2	General:	General:	General:
		Be understood; be helped (nurtured, given support)	Understanding (empathic, sympathetic), but ultimately not understanding (about symptom); accepting; respecting; open (expressive, available); controlling	Open (about symptom), not open (about himself); accepted; respected; comfortable
Parents	2	General:	General:	General:
		Be understood; be helped (nurtured, given support)	Not understanding; rejecting; don't trust me (don't believe me); dislike me (not interested in me); distant; unhelpful; strict (severe)	Not open (not expressive, distant)
Other person in general	2	General:	General:	General:
		Be understood; be helped (nurtured, given support), be opened up to; be open (express myself)	Helpful, but ultimately not understanding/ unhelpful (not reassuring, not comforting); out of control (unreliable) rejecting; distant; unhelpful; strict (severe)	Don't understand (confused, poor self-understanding); uncertain; unloved (alone); anxious; open (about symptom), not open (about himself)
		Typical:		
		Achieve		

#, Number of events; General, occurred in all events; Typical, occurred in more than half of the events; Wordings between brackets refer to “Standard category components” within the precursory “Standard category.”

In Step 3, researcher 1 calculated longitudinal intra-subject associations (i.e., correlations between two series of repeated measures within the same subject, in particular the questionnaire scores obtained at regular intervals throughout therapy, see “Procedure”) between evolutions in the patient's symptomatic and interpersonal level of functioning. Next, researcher 1 engaged in a “thick description” (Pontoretto and Grieger, 2007) of the longitudinal, clinical interplay between both levels throughout therapy, in which changes in quantitative measures were linked to the treatment narrative (Dattilio et al., 2010) and significant therapist interventions and extra-therapeutic events were discussed. Several precautions were taken to reduce researcher 1's biases and expectations, and to present a “truer” account of the data (see Hill, 2012): (1) prior to writing, researcher 1 orally presented her provisional analyses to a third research team member (familiar with the raw narrative material) and a colleague who was not involved in the research project (familiar with the theoretical orientation and phenomena of interest, and informed about the research questions). Both colleagues extensively questioned researcher 1 in order to focus findings and interpretations more clearly in response to the research questions; (2) during the writing process, researcher 1 continually returned to the raw material to stay close to the patient's narratives; included sufficient detail and literal quotes of the patient in the written document to validate presented findings; (3) the manuscript was reviewed several times by the team member and colleague described above, to identify areas in need of further attention, which were subsequently refined.

## Results

### Step 1: evolutions in symptomatic functioning

#### Analysis of outcome data

Figure 1 shows an overall increasing trend over the course of therapy (session 1–23) and during follow-up in both *self-reported* general psychological and physical functioning (as indicated by generally descending GHQ-12 and SCL-90 scores), and in *researcher rated* psychological, social and occupational well-being (depicted by generally increasing GAF-scores). When assessed by means of the Reliable Change Index, decreases in GHQ-12 values did not reach significance during treatment (RCI = −1.41, *ns*), but were significant when follow-up measures were included (RCI = −2.83, *p* < 0.05). In addition, large severity adjusted effect sizes (SAES) of changes were observed (d = 0.85 during treatment, *d* = 1.69 at follow-up). Decreases in SCL-90 values did not reach significance at follow-up (RCI = −1.66, *ns*) and a small severity adjusted effect size (SAES) was observed (d = 0.41). Noteworthy however, only three measurement points for SCL-90 could be obtained (at intake, at session 8, and at follow-up), considering that the patient lost the questionnaire set attached to session 16, and that session 24 was canceled because of no-show. The patient's reluctant stance toward active cooperation in therapy (see Results Step 3) also manifested in his nonchalant manner of completing (i.e., fast and monotonously, mostly marking the same answers every session) and handing over the questionnaires.

Several peak values can be noted during treatment. As addressed in Step 3, GHQ-12 scores—marking general distress—peaked during sessions 2, 7, 11, 15, and 20.

Next, Figure 2 depicts a variety of health care costs made in a period spanning from 2 years before the onset of treatment until follow-up. The top two graphs show that the patient's main health costs during that period went to (a) frequent consultations of his primary care physician and medical experts (especially dentists; see “Consultations” in Figure 2)—which were lowest *during* the treatment period—and (b) blood sample analyses, radiography and medical imaging (see “Blood Analyses & Medical Imaging” in Figure 2), which the patient never mentioned during the sessions. Importantly, during the observed period, no (medically prescribed) psychotropic medication was used, nor were there any periods of job absenteeism due to a physical or psychological condition. Moreover, despite the patient's intense fears of “terrible,” life threatening diseases, not a single hospital admission (day/residential/emergency hospital care) was administered. Every therapy session, the patient explicitly expressed his pride about not having consulted his primary care physician and not having taken any sedatives since the onset of treatment, which he greatly contrasted to the pre-treatment period.

The bottom graph of Figure 2 depicts the total sum of health care costs. In terms of average costs per month, a slight descending trend is observed from pre- to post-treatment. Costs were highest during the pre-treatment period (average of €27 per month) compared to the treatment period (€25 per month) and post-treatment (€23 per month).

#### Qualitative description of evolutions

Note: As we find it important to stay close to the literal wordings of the patient in illustrating our remarks, we frequently quote citations of the patient throughout the text, indicated by double quotation marks (“…”). Italics within these quotations indicate stressing by the research team.

At the onset of treatment, Chris complained of “anxiety or panic attacks,” arising from intense fears that certain bodily sensations (especially situated around the heart area) were omens of “terrible diseases” or precursors of sudden death. The self-declared “obsessive monitoring” of his own body (in the first place of “the normality” of his heart rate) consumed a lot of time and energy, caused him additional distress and intensified his “bodily symptoms”. Panic attacks occurred almost daily (sometimes amounting to several a day), varied in intensity and duration (from several minutes to several hours), took place in various contexts (see Results Step 3), and inhibited him in continuing his ongoing activities. Afterwards, he regularly felt exhausted.

Following the diagnostic sessions (see “Intake Phase” on Figure 1)—and thus “finally knowing what I suffer from”—Chris happily claimed to feel much better, and his symptoms diminished for several weeks. From the second therapy session onwards, however, “little attacks” started to rise again (see increase in GHQ-values in Figure 1) and he presented a new, “*psychological*” symptom, i.e., “being unable to speak about myself,” or “bottling up”. During the stable period (sessions 3–6) in which “the *bodily* aspect had almost disappeared,” this psychological symptom became “the biggest issue.” *Tipping point 1* (session 7) again yielded an upsurge in Chris' suffering (see second rise in GHQ-values, Figure 1): anxiety attacks were experienced more intense and sequenced each other more rapidly. In addition, Chris complained of intense agitation, felt tired and low-spirited, was “bottling up” again, and became very impatient concerning progress in therapy. Contrastingly, during sessions 8–10, he had “assumed a more acceptant attitude” toward his symptoms. This made him “feel stronger to ward off” his troublesome thoughts and fears, and “resulted in less frequent and less intense anxiety attacks.” Except for session 11, during which he complained of “very much heart problems” that occurred “without any reason,” this stable period of feeling “inwardly calm, less agitated” and being “more talkative,” prolonged until session 15 (see Figure 1). Yet, the stagnation in symptom frequency and intensity made Chris feel increasingly more frustrated and impatient. In session 15 *(tipping point 2)*, “bodily symptoms,” anxiety attacks and profound agitation were almost continually present and interfered intensely with his ongoing activities. This provoked a depressed, apathetic mood, uncertainty, and “a feeling of incapability to handle the future.” In contrast, sessions 16–19 again yielded increasing symptomatic and general well-being (see lowering GHQ-values, Figure 1). Chris generally felt “very good,” “much stronger,” and “less frightened in everyday life”; his thoughts “no longer continually lingered to hospitals and diseases”; anxiety attacks occurred only occasionally anymore and disappeared rather quickly; he talked more about himself, and looked brighter toward the future. Hence, following session 19, he interrupted therapy for 5 months. Upon return in session 20, panic attacks had re-appeared. Anew, he no longer felt “in control of my bad thoughts,” and complained of profound distress, low spirit and tiredness. As this condition ameliorated again during sessions 21–22, he asked to lower the frequency of therapy sessions. During the last session, he happily announced that his bodily aches and worrisome thoughts inhibited him increasingly less in daily activities, and that he felt “much more energetic and hopeful.” Although a new session appointment was scheduled, he did not return to therapy.

During the follow-up interview 3.5 years later, Chris affirmed maintenance of therapy gains: “I am feeling pretty good,” “I focus less on bodily sensations,” and “I have assumed the agitation” (that he still tried to ward off during the treatment period) “as part of who I am.” “Good periods” continued to be interrupted by “temporary dips,” during which “built-up tensions broke out physically,” anxiety attacks occurred more often, and anxiety, tiredness and anger were more present. However, he had “learned to live with the anxiety attacks, and cope with them without resorting to medication.”

### Step 2: evolutions in interpersonal functioning

#### Analysis of outcome data

Figure 3 depicts the evolutions, throughout therapy and during follow-up, in both IIP-32 total values and scores for the dependent and autonomous IIP-32 sub-profiles. Similar to Figures 1, 3 shows a generally descending trend in IIP-32-scores (i.e., decrease in reported interpersonal problems) throughout therapy (session 1–23), which reaches significance when assessed by means of the Reliable Change Index (RCI = −2.47, *p* < 0.05) and which corresponds with a large severity adjusted effect size (SAES; *d* = 0.99). During follow-up, this decrease is maintained.

Overall during therapy and follow-up, scores for the dependent sub-profile are higher than for the autonomous profile.

Several peak values (i.e., increasing interpersonal problems) can be noted during treatment. As addressed in Step 3, IIP-32 scores peaked during sessions 4, 6, 9, and 16.

#### Analysis of CCRT-codings

Tables 1–4 present CCRT-ratings of RE's with specific people and the other person in general, during the first therapy sessions (Table 1), around *tipping point 1* (Table 2), around *tipping point 2* (Table 3), and during the last therapy sessions (Table 4). Before discussing the main findings in the next paragraphs, two preliminary observations regarding the patient's interpersonal functioning are noted.

**Table 2:** Patient's wishes (W), responses of other (RO), and responses of self (RS) in sessions 5–7.

**Target of interaction**	**#**	**W**	**RO**	**RS**
Anna	1	General:	General:	General:
		Be understood; be helped (nurtured, given support); be close; help (give to); be good (do the right thing, be perfect); have control	Understanding; accepting; respecting; open (expressive, available); loves me; controlling	Open; not open; accepted; respected (valued); like her; helpful (try to please, giving); dependent; comfortable (safe, secure)
Ex-girlfriend	1	General:	General:	General:
		Be respected; be close to; be loved; not be hurt	Controlling; not understanding; rejecting; dislike me (not interested in me); distant; not trustworthy	Dependent; uncertain (ambivalent, conflicted); disappointed; unloved; depressed; helpful
Parents	1	General:	General:	General:
		Be understood; be helped (nurtured, given support)	Not understanding; rejecting; don't trust me (don't believe me); dislike me (not interested in me); distant; unhelpful; strict (severe)	Not open
Other person in general	7	Typical:	Typical:	Typical:
		Have control (have things my own way); be independent; hurt (get revenge)	Controlling; don't understand; angry	Angry (resentful, irritated); hurt others (hostile); controlling (dominating, aggressive); dependent; symptom (anxious, somatic complaints); not open
		Variant:	Variant:	Variant:
		Be understood; be helped (nurtured, given support), be opened up to; be open (express myself); be close (not to be left alone)	Not understanding; out of control (unreliable); helpful	Anxious; unloved (alone)

#, Number of events; General, occurred in all events; Typical, occurred in more than half of the events; Variant, occurred in at least 2 events; Wordings between brackets refer to “Standard category components” within the precursory “Standard category.”

**Table 3:** Patient's wishes (W), responses of other (RO), and responses of self (RS) in sessions 14–17.

**Target of interaction**	**#**	**W**	**RO**	**RS**
Anna	5	Typical:	Typical:	Typical:
		Be understood; be helped (nurtured, given support); be close	Controlling; angry (irritable); loves me; understanding; accepting; respecting; open	Not open; angry (irritated; resentful); anxious; dependent; comfortable (safe, secure); loved
Parents	2	General:	General:	General:
		Be understood; be helped (nurtured, given support)	Not understanding; rejecting; don't trust me (don't believe me); distant; unhelpful; strict (severe)	Uncertain; anxious; distant; disappointed
Other person in general	3	General:	Typical:	General:
		Have control (have things my own way); be independent; feel good about myself (be self-confident)	Don't trust me (don't believe me); not understanding; not respectful; rejecting	Distant; not open
				Typical:
				Ashamed

#, Number of events; G, General (occurred in all events); T, Typical (occurred in more than half of the events); Wordings between brackets refer to “Standard category components” within the precursory “Standard category.”

**Table 4:** Patient's wishes (W), responses of other (RO), and responses of self (RS) in sessions 21–23.

**Target of interaction**	**#**	**W**	**RO**	**RS**
Anna	4	General:	General:	General:
		Be understood; be helped (nurtured, given support); be close; help; achieve; better myself	Loves me; understanding; accepting; respectful; open	Comfortable (safe, secure); loved; open
				Typical:
				Anxious
Parents	5	General:	General:	General:
		Be understood; be helped (nurtured, given support); be close (not left alone)	Not understanding; rejecting; don't trust me (don't believe me); distant; unhelpful; strict (severe)	Uncertain; anxious; distant; disappointed
			Variant:	
			Dislike me (not interested in me)	
Ex-girlfriend	2	General:	General:	General:
		Be respected; be close to; be loved	Controlling; not understanding; rejecting; distant; not trustworthy	Disappointed; unloved; depressed; angry (resentful)
Other person in general	6	Typical:	Typical:	Typical:
		Be understood; be helped (nurtured, given support); be close (not left alone); be respected; achieve; compete	Not understanding; rejecting	Distant; not open; ashamed; hurt others (hostile); self-confident
		Variant:		Variant:
		Assert myself		Unloved (alone); disappointed; angry

#, Number of events; General, occurred in all events; Typical, occurred in more than half of the events; Variant, occurred in at least 2 events; Wordings between brackets refer to “Standard category components” within the precursory “Standard category.”

The first remark concerns the patient's characteristic manner of narrating in a very abstract manner about relations. Throughout the entire therapy, interpersonal references are scarce in the patient's discourse and are, without exception, reported as general accounts of typical, context (i.e., time and place) independent relational exchanges, which are never linked to specific (past or current) events. This is reflected in Tables 1–4 by the dominance of “general” W's, RO's, and RS's. The patient typically speaks about the other person in terms of “they,” “my fellow man,” or “it” (e.g., “it was very crowded at the checkout”), without specifying concrete individuals. Specific others (even the patient's partner and his so-called “key persons,” i.e., his closest friends, who are never mentioned by name) are never described in terms of character, of what attracts him in them, of what he dislikes about them, of common interests or recurring conflicts, etc. Concrete everyday examples of relational exchanges are never rendered spontaneously, and only reluctantly when asked for by the therapist. Correspondingly, Table 1 shows that narrated RE's during the first therapy sessions are limited to 6 (instead of the required 10 for further CCRT-coding, see Method).

Second, the therapeutic relationship is not delineated separately in Tables 1–4 due to absence of clear CCRT-components in the enacted interactions during therapy. However, it proves significant with regard to the patient's interpersonal functioning. Overall, the therapeutic relation is markedly characterized by a hesitancy of the patient to cooperate in the therapeutic work. The patient expresses this by occasionally arriving late in sessions; forgetting questionnaires; frequently communicating his annoyance with the continuing absence of therapeutic progress and his dislike of “talking about myself”; persevering in a strict focus on his symptoms; answering very briefly and dismissively to questions; repeatedly interrupting the therapist in a loud tone; and extensively reporting the results of his daily quests for alternative ways to get better (e.g., by acupuncture, herbal medicines, etc.).

#### CCRT's in RE's with specific others

##### With anna

Throughout the entire therapy, Chris' girlfriend Anna remained the only person with whom he felt “at ease” enough to “be myself.” Whenever he faced rising anxiety levels, he immediately informed her. For him, their interactions predominantly served the purpose of reassuring him during or after anxiety attacks: “her job” was to tranquilize his fears by “rationally contradicting” the likelihood of their underlying cognitions. He generally experienced Anna as complying with these wishes, which made him feel good and safe. Yet, as she “had never experienced anxiety attacks personally”, she would “never be able to truly understand” what he lived through every day, and he was “not able to explain it properly.” During therapy, her demand to “talk more about myself” (i.e., “to say what I think and feel”) aroused increasingly more irritation in him, and did not incite him to strive toward meeting this wish. As therapy proceeded, Chris increasingly became aware he had “never been treated with so much love” and, consequently, was “afraid to lose it again” by “doing something wrong that would make her angry and want to leave.” Out of this self-declared “fear of failure,” he tried to put his best foot forward toward her. Near the end of therapy, he spoke increasingly more in terms of “dependency” and “separation anxiety” in relation to her, which he was ashamed of admitting, as it collided with his ideals of being “strong” (i.e., “not needing anyone”) and independent.

##### With parents

Throughout the entire therapy, Chris solely disclosed about past interactions with his parents, to illustrate the sharp contrast between their and Anna's stance toward him as being “the complete opposite.” Whenever he had consulted his parents in the past with fears about bodily sensations, and had wanted to be empathized with and taken care of, they had “never taken him seriously,” but had “always laughed off” his concerns. In addition, they had always “fixated” on his failings and had never shown any signs of genuine interest (e.g., “my father did not even know what school year I attended”) or “overt love” (e.g., hugging, kissing, complementing). As a result, Chris had since long refrained from confiding anything in them.

##### With ex-girlfriend

Occasionally appearing in his discourse throughout therapy (see Tables 2, 4), Chris' ex-girlfriend was (similar to his parents) contrasted to Anna as the rejecting and uninterested person who had “caused me much harm.”

#### CCRT's in RE's with other person in general

During the first half of therapy (see Tables 1, 2), most narrated RE's concern Chris' so-called “fellow man” in general, whom he never concretized or described as specific individuals. Especially in the first and last therapy sessions (see Tables 1, 4), Chris expressed, out of a feeling of “being alone in the battle,” a profound desire to find a “fellow sufferer, who understands what it is like, who lives through exactly the same as I do” (session 2); a daily quest in which he invested a considerable amount of time, but without satisfying results. Around the first and second *tipping points* (see Tables 2, 3), Chris' “fellow man” started to appear as someone whose daily expectations—or even appearances—did not fit into his well-structured time schedule, someone with whom he had little patience, and who “agitated” him because of his/her “slowness”. At the start of every day, he “pictured an image of the perfect day” and whenever something came in between (i.e., slow traffic, long rows at the supermarket, receiving a new deadline at work), he felt “annoyed” and “took revenge” by becoming irritable, “short-spoken” or downwards aggressive (i.e., by loudly blowing the horns of his car). From session 9 onwards, others also appeared as important sources of criticism, “confronting” him with his flaws and mistakes and inducing a sense of threat, impotence and shame. Others' expected reactions of “incomprehension” (e.g., “don't clown around,” “get yourself together, be a man”) in response to his symptoms, were the main causes for “hiding” his anxiety attacks for “the outside world,” and for adopting a “mask” or “pretense” of “the cheerful, assertive, independent man” who thrived in a “highly competitive, capitalistic job.” Hence, depicted W's to be helped (which are so characteristic for RE's with Anna) merely refer to past, frustrating RE's with others, especially during his first anxiety attack in Dubai.

### Step 3: associations between symptomatic and interpersonal level

#### Analysis of outcome data

In line with expectations, longitudinal intra-subject correlations between IIP-32 scores and GHQ-12 scores document a positive association between the patient's interpersonal and symptomatic functioning throughout therapy (*r* = 0.35, *ns*). However, the observed correlation did not reach significance. Due to the small number of measuring points, longitudinal intra-subject correlations between IIP-32 scores, on the one hand, and SCL-90-R and GAF scores, on the other, were not calculated.

#### Qualitative description of associations

In this part, we contextualize the outlined symptomatic and interpersonal changes (see Step 1 and 2) in descriptions of the dynamics of the treatment process, specifically focusing on the interactions between both. The influential impact of both therapeutic and extra-therapeutic factors or events is discussed. With respect to therapist interventions, we specifically refer to the conducted manual of supportive-expressive psychodynamic treatment (Luborsky, 1984) by italicizing concrete interventions, followed by their designation as “expressive technique” (“ET”) or “supportive technique” (“ST”) and the related page in the manual. Literal wordings of the patient are indicated by double quotation marks (“…”).

Chris entered therapy with complaints of daily occurring “anxiety attacks” or “panic attacks” that had abruptly started 4 months prior to the onset of treatment. The first, most intense one, had “suddenly” and “without any reason” occurred “while being alone” on a business trip in Dubai, whereas countless previous business trips had always come about successfully. Back home, however, a “quiet,” symptom-free period of 2 months had succeeded, before a second, equally unexpected, attack had occurred, this time in the presence of his girlfriend Anna. Since then, anxiety attacks had “overtook” him on a daily basis, in fluctuating intensities and durations, and in a variety of contexts, between which he dispiritedly discerned “no link whatsoever.” Hence, as the attacks “could strike me anytime, anywhere,” he continuously experienced “anticipatory anxiety” and felt intensely “uncertain.” In marked contrast with “the assertive and strong man I was before,” they divided his subjective experience in “before” and “after” the start of the attacks. Ashamed, he scrupulously hid the attacks for “the outside world,” secluding himself from the surrounding people whenever he felt them emerge.”

The recurrence in anxiety attacks had prompted him to consult his general practitioner, who had subsequently referred him to psychotherapeutic treatment. However, initially reluctant to acknowledge that “something psychologically could be involved” or that “talking would somehow help,” he had waited a month to consult the therapist, finally incited by Anna, who was convinced his attacks resulted from his “non-talkativeness,” i.e., “bottling up my feelings and thoughts” (session 2). *Encouraged by the therapist to elaborate on this subject (ST, p.87, p.89*; *ET, p.94*; session 2), Chris indifferently shrugged off Anna's comment by saying “I am simply unable to talk about myself; I dislike it and I have never learned it at home, we never shared feelings, thoughts or opinions with one another.”

Accordingly, during initial therapy sessions, Chris's speech was marked by continuous recitals of perplexing symptomatic appearances, often merely listing up the frequency and intensity of attacks over the past week, without any additional context (i.e., unrelated to any preceding or subsequent incidents/emotions/thoughts/reactions/interactions). *In response, the therapist repeatedly incited Chris to illustrate his remarks with concrete examples (ST, p.87, p.89; ET, p.94; sessions 1-3)*, to which Chris initially was profoundly reluctant, muttering he did not understand why the therapist had to “drag all these things into” therapeutic discussion. In addition, with each provided example, Chris firmly stressed that the “bodily sensations” he felt were “real” (i.e., “not imagined”), but that subsequent, “psychological” fears and thoughts (i.e., of suffering from “acute heart failure” resulting in his “sudden death”) made him “exaggerate” these sensations. This initiated a “vicious circle” of mounting anxiety “from which there was no escape.” In this context, he had experienced his general practitioner's referral to “a psychologist” as deeply insulting, feeling he had “not been taken seriously.” Hence, his therapy aim was to acquire effective strategies to deal with these exaggerating thoughts, in order to limit the anxiety attacks. In the meantime, however, he remained convinced that “the only thing that could possibly help me” was “to talk to a fellow sufferer,” i.e., someone “who goes through exactly the same as I do, for only this person would be able to truly understand me” (session 2). Accordingly, he daily consumed hours of time on Internet forums in pursuit of such a person, scanning the experiences and coping tips shared by other anxiety sufferers. Yet, as Chris experienced each of these persons to differ in one way or another from himself, he did not readily find his counterpart.

*As the therapist drew attention to (ET, p.121; session 2)* the great importance Chris attached to “being understood” and “being taken seriously” by others, Chris immediately appended that “the absolute worst thing” about his first panic attack abroad was that “nobody spoke my mother tongue.” In fact, at the height of his attack, he “had almost sent a text message to Anna.” Promptly, *the therapist linked this pronunciation with previous phrasings (ET, p.94, p.118, p.131; ST, p.89*; session 2) concerning his symptom, in which Anna's involvement was also apparent (e.g., “Anna and me call them ‘my little attacks’,” “me and Anna are reading up on obsessions,” session 1) and consequently *pointed to the marked contrast (ET, p.110, p.118; ST, p.89*; session 2) between this “appeal” and his inclination to hide the attacks from everyone else. Chris added in assent that he “reported” each attack to her (i.e., on the phone or when arriving home after work), because “her job is to reassure me” by “logically remonstrating each of my cognitions and clearly showing me why it is highly unlikely that I suffer from a terrible disease or heart failure.” He clarified that he recognized the “absurdity” of his fears, seeing his young age (i.e., 26 years) and a complete absence of familial predisposition for heart disease, and continued laughing that his habitual smoking behavior and unconcern for exercise or healthy food collided markedly with the described fears. However, during an anxiety attack, he was always “completely convinced” of “their verity.” *When further asked to expound (ST, p.87, p.89; ET, p.94*, session 2) on his tendency of addressing Anna with his symptom, Chris emphasized that the attacks were “the only thing I cannot properly explain to her” and “the only thing that do not fit into the otherwise perfect relationship.” “And the timing is not right,” he added, “I really do not understand why the attacks started nòw. Now that I am finally at èàse, and for the first time engaged in a gòòd relationship, thìs comes along. It is as though all the misery of the past 20 years has all of a sudden burst out now.” *At the therapist's incitement (ST, p.87, p.89; ET, p.94*, session 2), Chris clarified the sharp contrast between his current relationship with Anna, on the one hand, and past relations with his parents and ex-girlfriend, on the other, as being “the complete opposite,” a theme that was frequently resumed throughout the following sessions. Whereas the latter had “always fixated on my weaknesses,” “dismissed my accomplices as common or normal” and had never shown any genuine interest in him (e.g., “my father did not even know what school year I attended”), “Anna pointed out what I am good at” and did prompt him to share his thoughts, feelings and opinions with her. Yet, he experienced this “demand” as “annoying,” and habitually passed it over with the pretext of “I don't know what to say,” “I simply don't know how,” “What does it matter what I think at the moment?” or “It is too tiresome to try and put it into words.” For he stubbornly contradicted Anna (and the therapist) that his anxiety attacks could be connected to this “non-talkativeness.”

However, as the initial acuteness of his symptoms diminished (see stable GHQ-12 and SCL-90 scores in Figure 1), Chris gradually became more receptive to *the therapist's interruptions of his persistent recitals of symptomatic appearances, to expound more on interpersonal references present in the provided examples (ET, p.94, p.131)*. During sessions 3-5, his non-talkativeness became a more prominent theme of discussion (see increasing IIP-32 scores in Figure 3) and from session 5 onwards, Chris started to name the “biological” and “psychological aspect” in one breath, e.g., “I'm feeling better and I am also talking more about myself to Anna,” “Maybe I used to talk too little about my feelings, corked them up too much, and that is why all the stress condensed on my body.”

Together with this gradually increasing focus on (inter)personal issues, for the first time since the start of therapy Chris' “fellow being” (i.e., the other in general, see Results Step 2) appeared in his speech. He started session 5 declaring: “Now it is particularly when I am feeling agitated, that I suffer from anxiety attacks. And (laughing) I am very easily agitated.” *Encouraged by the therapist to illustrate this diffusely termed “agitation” (ST, p.87, p.89*, session 5), Chris provided miscellaneous examples, *from which the therapist deduced (ET, p.98, p.118)* they all had something to do with “being impatient” and suddenly “wanting to get away” from places (i.e., traffic, his office, the supermarket), but being hampered in this by others. *As the therapist further underlined the contrast (ET, p.110, p.118; ST, p.89*) between this impatience to get away from others and his previously demonstrated appeal to Anna, Chris expanded on his wish to be close to her. He disclosed he “had never experienced so much love in his life” and that he was “utterly afraid to lose it again” by “doing something wrong that would upset her and make her want to leave” (see also Chris' elevated score on the IIP-32 subscale “Intrusive,” reflecting his fear to be alone, i.e., without Anna). This engendered a newly acknowledged discrimination between two “types” of fear he had previously assembled together: on the one hand, his fear of not being able to please Anna sufficiently; on the other, the fear induced by unwelcome intrusions of all others in his well-organized time schedules. The intense sore throat, with which Chris entered session 6, further brought the subject round to time schedules, obligations and duties. Loudly complaining about the upcoming wedding celebrations of a close friend which he “hàd to attend, I have no other choice”, Chris articulated in one breath: “I am afraid of being seriously ill ànd of not doing what is expected of me.” *Asked to expound on this “time schedule” (ST, p.87, p.89)*, Chris disclosed about his habit to start the day off envisioning “an image of the perfect day,” in which he always wanted “to have the final decision” (“It is all about control, I want to have control”; see also his elevated score on the IIP-32 subscale “Domineering”). Whenever something unforeseen occurred (which inevitably happened multiple times a day, e.g., slow traffic, crowded super market, additional work deadline), he always felt his temper rise. *As the therapist tied this up with previous disclosures about “not expressing, but bottling up his feelings” (ET, p.94, p.110, p.118, p.131; ST, p.89)* and further inquired about the precise contents of the latter, it began to dawn on Chris that his corked up frustration not only caused him to “explode at some point” (i.e., reacting coldly or aggressively to others), but also incited him to contract his muscles, which eventually provoked pain, i.e., the “real, not imagined” pain he so strongly emphasized since the referral of his general practitioner. For the first time during treatment, he explicitly praised the value of focusing therapeutic discussion on contextual elements, beyond the strict symptom: “At first I was very skeptical of your way of working, I often thought ‘What does that have to do with anything?’ but now I can see the point of it.”

Moreover, *intensifying therapeutic focus* (*ET, p.94, p.110, p.118;* sessions 5–8) on others' intrusive obligations as colliding with his preferred timetable, during sessions, further incited climbing agitation levels outside of therapy (see temporary increase in reported autonomy problems in Figure 3, and simultaneous decrease in dependency issues [IIP-32-dep]), and accumulated in peak scores in general and symptomatic ill-being in session 7, *tipping point 1* (see Figure 1). *Time after time incited by the therapist to talk this agitation through (ST, p.87, p.89)* during sessions 8–10, while discriminating it from the love and ease he felt with Anna, Chris' peak scores dropped again (see Figures 1, 3) and he reported to “feel much calmer”. However, in sessions 10–11, transferential impatience started to mingle with therapeutic progress: Chris reverted to repetitive reports of symptomatic flares—that rose anew in session 11 with “an awful lot of heart problems” (see Figure 1)—while complaining again about the monotony of the sessions, which crept unwelcomly into his tight schedule. Yet, *the therapist insistently cut across Chris' repetitive discourse, by paraphrasing (ET, p.94, p.114, p.118; ST, p.89)* Chris' previous remarks about “being impatient to get awày fròm” into the inquiry whether his first anxiety attack abroad had possibly been preceded by “an impatience or eagerness to get bàck tò Anna.” In response, Chris elaborately opened up about “that first time in Dubai,” adding new elements to his previous fragmented narratives that shed new light on the start of his symptom. This business trip had been the first one right after he had moved in with Anna. Opposed to all previous trips, he had—for the first time ever, as he used to be a perfervid traveler—dreaded “leaving her and going away from home.” *In reply to the therapist's prudent suggestion (ET, p.94, p.98, p.114, ST, p.89)* that (the creation of) his symptom had thus granted him with an excuse to return home earlier, Chris shamefully admitted “This is the first time I prefer being home above anywhere else, whereas I have always been so eager to leave.” “The farther she is, the worst I'm feeling.” Although he initially framed this experience negatively as “being overly dependent” and “maybe it is not healthy” (reflected in the overall higher IIP-32 dependent profile, compared to the autonomous profile, in Figure 2), he declared in session 12 to “feel much calmer” again, and “it was an interesting session last time.” Synchronously, he pushed forward a new therapeutic endeavor: “It is all about learning to analyze my feelings, is it not, which I have apparently never done before, learning to cope with frustration and sorrow and uncertainties,” and anew showed a greater willingness to answer therapeutic questions about issues beyond the mere symptom. Consequently, in reply to a question concerning the renovation plans that kept him busy in session 12, Chris uncovered a new element relating to the start and the following persistent recurrences of his symptom. Whilst moving in with Anna (1 week before his business trip in Dubai), in the former house of her parents, in which many objects still evoked memories of the latter, he had found out Anna's father had very unexpectedly died at the age of 27 (i.e., 1 year older than he currently was) of a heart attack. Like in his own family, there had been no familial predisposition for heart disease and “he had optimal cholesterol values.” *As the therapist's referred to (ET, p.94, p.118; ST, p.89)* previous remarks concerning Anna's job of reassuring him “that nothing is going to happen,” Chris added in assent: “And that is exactly why Anna can never guarantee that nothing is going to happen: the proof is her father!”

Next, following a relatively stable period (session 13–14, see also Figures 1, 3) in which the same themes were further worked through, a profound extra-therapeutic event preceding session 15 (*tipping point 2*) abruptly urged for a turn in Chris' current way of addressing Anna via his symptom. Anna had assured Chris she “was fed up” with him needing help all the time. “Even when she is not feeling too well herself, she still has to take care of me; she should also have the right to have a bad day.” Utterly afraid of “ruining precisely what you, above all, certainly do not want to be ruined” (session 15), anxiety levels (see Figure 1) and reported interpersonal troubles (see Figure 3) temporarily rose again.

Remarkably, from session 16–19, Chris started to report important transformations, both symptomatically and (inter)personally (see decreasing trend in Figures 1, 3). He described to be “less focused on my body” because “I know perfectly well it is not true [NB: what he fears is going to happen],” as a result of which anxiety attacks occurred less intense and less frequent. He had managed to peacefully go on a short business trip “without any problems” (session 17). As Anna had “broken through the wall I had built around me” Chris increasingly “involved her in important decisions” instead of “deciding everything on my own, like I had always done” and stopped time-consuming pursuits of “a fellow sufferer.” *Upon the therapist's appraisal of this therapeutic gain (ST, p.86;* session 16), Chris low-spiritedly added that the “encounter of so much love” had also woken him up to the fact that “I am not the strongest person in the world anymore.” “That is the price we pay to let someone into our lives.” Next, *as the therapist took up Chris' phrasings (ET, p.94;* session 17) of Anna being “the only one with whom I can be myself and drop the pretense,” Chris continued he hid his symptom out of “shame” and “the fear of being accused of play-acting and seeking attention.” *Upon the therapist's inquiry after his parents' reactions when he had been ill as a child (ET, p.110, p.118; ST, p*.89) Chris reminisced they had always coldly brushed aside his wailings (i.e., “no yammering, do not look at the spot where it hurts and it will blow over”), and “never took me seriously.” *When the therapist linked this latter phrasing to Chris' indignation about his GP's referral to psychotherapy (ET, p.94, p.118, p.131; ST, p*.89), Chris particularly recalled a vivid memory of their disbelieving reactions to “what they thought was just a regular cold, but which eventually turned out to be a terrible pneumonia for which I had to be hospitalized,” Markedly, *as the therapist's further inquired after Chris' habitual reactions to his parents' disregarding behavior (ET, p.110, p.118*), he described how he had become hyper alert for bodily signals, “always dreading there was something wrong with me.” *Again egged on by the therapist to elaborate on these themes (ST, p.87, p.89; ET, p*.94) throughout the following sessions, Chris started to realize “that this is the reason why I tend to act aggressively toward others, as a way of immediately asserting myself and showing I am not to be crossed with, that I am to been taken seriously.” For he usually anticipated critique and rejection of others, but “I do not know how to react. I'm thinking a lot, but I do not say anything.”

Having thus far progressed in therapy to permitting himself not to be as “strong” as he “used to be” or as he wished to display to the outside world, but to be “dependent of someone,” and ask for and accept Anna's care, Chris' symptomatic, general and interpersonal wellbeing steadily increased over sessions 16–19. He and Anna heartily made plans to start a family, and Chris stopped attending therapy after the 19th session. A 20th session had been scheduled, but Chris failed to show up.

However, 6 months later, a second extra-therapeutic event triggered a new destabilization in Chris' life that again urged for a change in interpersonal stance, inciting him to consult the therapist anew. The imminent birth of his first child with Anna urged to confine his recently admitted dependency to a certain extent, in order to “be able to provide care myself.” As he yearned “to be strong enough again” so that “Anna will not have to take care of three all by herself,” he wished to attend therapy “preventively.” For he feared anxiety attacks would start rising again as long as he had not learned to “cope with uncertainty and anxiety and feelings all together” but still tended to express them “in a physical way.” Moreover, reminiscent of the abrupt death of Anna's father when she had still been a toddler, he added: “Now I will be the father and I'm terrified something will happen to me.” *As the therapist asked for Chris' interpretation of “being a father” (ST, p.87, p.89)*, Chris immediately emphasized he and Anna certainly did not want to raise their child like his parents had done, but “in a much more physical way” (i.e., with more loving physical contact, e.g., hugging, kissing, etc.) and “more open-faced, less coldly.” In the following sessions (21–23), *the therapist mainly took on a supportive, incentive stance (ST, p.87)* while Chris mainly resumed illustrations of his parents' and ex-girlfriend's rejecting behaviors, in opposition to Anna's stance toward him, and his own desired stance toward his future child. Notably, he talked these themes through in a much more elaborate and calm, reflective way (see decreasing ill-being scores in Figures 1, 3). More fiercely, he reminisced past encounters with a group of friends who had “exploited” his generosity and “did not return it” when he had needed their friendly support. At the end of session 23, he concluded: “I have grown from a general ‘people pleaser’ to a hot-tempered person who is very talented in his capitalistic job” and “who prefers to spend time with his family,” for they are “the only ones who deserve it.” *When the therapist finished the session “with this admirable character sketch” (ST, p.86, p.89)*, a new appointment was scheduled, but Chris did not return to therapy.

## General discussion and conclusion

The present study started from symptom specificity as a fundamental concept in psychodynamic theory. In this context, Blatt's classical symptom specificity hypothesis (Blatt, 1974, 2004) describes differential associations between specific types of neurotic symptoms and specific styles of interpersonal functioning. Earlier nomothetic research into these associations, however, yielded mixed results, which have been argued to be due to several methodological and conceptual limitations inherent to cross-sectional group designs (see Cornelis et al., in press). In an effort to enhance a richer understanding of symptom specificity and to detect areas where potential *refinement* of Blatt's hypothesis proved necessary, the present empirical case study aimed to contribute to *theory building* (Stiles, 2009) in a clinically meaningful way (e.g., Edwards et al., 2004). A specific combination of Consensual Qualitative Research for Case studies (CQR-c; Jackson et al., 2011), and the “Core Conflictual Relationship Theme” method (CCRT; Luborsky and Crits-Cristoph, 1998) was applied to investigate dynamic associations between a patient's obsessional symptoms and interpersonal characteristics throughout a longitudinal therapy process. This research method has previously been developed and applied in a similar theory-building, empirical case study of a patient with obsessions (see Cornelis et al., in press). In the current paper, concrete predictions based on the *classical symptom specificity hypothesis* were contrasted to *alternative predictions* based on the findings of this previous study.

In line with expectations based on the classical symptom specificity hypothesis, the longitudinal intra-subject correlation affirmed a positive association between the patient's symptomatic and interpersonal functioning throughout therapy. Although the correlation did not reach statistical significance, extended qualitative analyses of the narrative material affirmed *close associations*. Despite the patient's initial reluctance to expand on the specific contexts in which his symptoms occurred, interpersonal components proved to be present in the cited examples, asked for by the therapist (e.g., the patient reported each and every symptomatic occurrence to his partner, while simultaneously segregating himself from all other persons). In accordance with findings from previous studies (e.g., Grenyer and Luborsky, 1996; Luborsky and Crits-Cristoph, 1998; Slonim et al., 2011), repeated therapeutic incitement to elaborate on these interpersonal associations (as supportive-expressive therapy prescribes) revealed significant past and present contexts. Throughout therapy, recurrent articulation of these contexts progressively elucidated linkages between the patient's tendencies to bottle up irritations toward others, on the one hand, and the start of and evolutions in symptom frequency and intensity, on the other. This process gradually shed light on underlying, frustrated wishes toward others (and its transference in the therapeutic relationship), and on the particular function of the patient's symptom within significant relationships. The gained insight incited the patient to gradually occupy different interpersonal positions within these relationships, and to live up to—previously unrecognized—wishes in a non-symptomatic way, which progressively rendered the function of his symptom redundant. In addition, the therapist's repeated efforts to engage the patient in a joint search for understanding (Luborsky, 1984) constituted new, unfamiliar relational exchanges that satisfied the patient's profound wishes to be understood and to take seriously. The patient's subsequent sense of a helping alliance and of therapeutic progress increased his involvement in therapy, and lessened his fierce quest for a fellow sufferer. However, at times these changes also raised fears and accompanying resistances that impeded him in the realization of these wishes, and induced temporary symptomatic increases. Throughout the gradual decomposition of old characterizations (during the main part of the therapy) and construction of new identity parts (during the final sessions), the patient progressively managed to organize the symptom's determinations in a coherent story, thereby recounting initially enigmatic experiences in meaningful structures. In line with expectations, this process curtailed the initially fierce ambivalences. Diminished struggles with his needs toward closeness (i.e., to his loving partner) *and* control (i.e., over unreliable others) finally culminated in a new character sketch, which united two irreconcilable tendencies in a newly gained sense of conformity.

In contrast to the symptom specificity hypothesis, but in accordance to previous findings (Cornelis et al., in press), self-reported interpersonal problems proved to be higher for the *dependent* than for the autonomous sub-profile. Further in line with prior results, CCRT-analyses revealed in addition to the predicted *autonomous* CCRT-components, persistent *dependent* W's to be close to significant others and RS's aimed at gaining their love and desired proximity.

However, while both the previous and current case study of obsessional patients reported co-occurrences of autonomous and dependent CCRT-components, an important point of difference can be noted between the findings. Cornelis et al. (in press) observed these co-occurrences *within each* relationship of the patient under study. Exaggerated tendencies toward autonomy and self-definition proved to be a means of dealing with profound dependent struggles toward all significant others. However, as autonomous strivings were typically accompanied by intense fears of losing others' love, the patient experienced profound *ambivalences* between dependent and autonomous behavior. Subsequently, ambivalence manifested on two levels. On the one hand, repeated alternations between appealing and repelling behavior toward others manifested *within each* relationship. On the other, ambivalence was expressed in intermittent alternations in choosing *between two* equally important relationships he experienced as irreconcilable.

For the current patient, discord between dependency and autonomy also occurred on two levels, but in a different way. First, it was most pronounced in the patient's strict division *between* his current romantic and all other relationships. From the onset of therapy, the patient radically distinguished close and satisfying exchanges with his girlfriend from mutually rejecting interactions with all other people. Central in *all* cited exchanges were wishes to be understood and respected in his “bodily” suffering, and to be helped during panic attacks. In addition, however, interactions with his girlfriend were highly characterized by *dependent* CCRT-components, which did not occur in encounters with others; while the latter revealed profound *autonomous* elements, which were absent in the first-mentioned. Appealing, devoted behavior, on the hand, and aggressive, repelling behavior, on the other, did *not* alternate *within* relationships, but were strictly divided *between* relations and relatively constant within each relationship. Qualitative analysis of the narrative material showed that the patient's obsessional symptom (i.e., panic attacks) functioned as a means of *addressing* his girlfriend in provoking her *care*; while it permitted him to *separate* himself from and install a sense of *control* over all others.

Hence, in contrast to what Cornelis et al. (in press) observed, *ambivalence* did *not* manifest in pressing urges to *choose between* equivalent relationships, nor in capricious behavior *within* each relationship. It did, however, manifest in the patient's difficulties to reconcile two “parts” of his identity he experienced as opponent. Whereas he had always known himself to be a “people pleaser” toward everyone, both his late aggression toward others, and his so-called “overdependence” toward his current girlfriend, startled him. The shame for these recent dependency issues made him hide behind a mask of “the assertive, independent man” he displayed to the outside world, and explained the overall higher dependent IIP-32 sub-profile.

Second, *ambivalence* manifested *within* the relationship with his girlfriend, the only one he found important. At the backside of his over-reliance on her, he also hedged himself against it by stubbornly dismissing her most profound wish toward him, i.e., talking more about himself. Repeatedly throughout therapy, *ambivalence* between highly dependent wishes toward closeness, and autonomous tendencies to ward off a feared intrusiveness from her, caused tensions. The presence of multiple interpersonal themes rather than a single predominant core, has previously been reported by, e.g., Crits-Christoph et al. (1994). Further consistent with previous findings (e.g., Crits-Christoph and Luborsky, 1990; Wilczek et al., 2004; Vinnars et al., 2013; Cornelis et al., in press), the patient's CCRT's did not change substantially throughout treatment, but increased awareness of the different wishes accompanied more flexible (i.e., less symptomatic) ways of living up to them. In line with expectations, these changes were accompanied by transformations in symptoms, as previously evidenced by, e.g., Cornelis et al. (in press), Crits-Christoph and Luborsky (1998), Grenyer and Luborsky (1996), and Slonim et al. (2011).

Accordingly, self-report questionnaire scores and the patient's narratives demonstrated significant improvements throughout therapy, both in the patient's symptomatic and interpersonal functioning, as in his general well-being and health care consumption. In accordance with results from Randomized Controlled Trials and other large-scale studies on the efficacy of psychodynamic therapy (see recent reviews of Fonagy, 2015; Leichsenring et al., 2015), improvements were maintained at follow-up.

## Conclusions, limitations and future research indications

The present empirical case study aimed to address several conceptual and methodological limitations intrinsic to statistical hypothesis-testing research in cross-sectional group designs, in an effort to further enhance a rich understanding of symptom specificity. In accordance with previous findings (Cornelis et al., in press), the study did not report a mere interpersonal tendency toward *autonomy* in the obsessional patient, but documented profound *ambivalences* between dependent and autonomous interpersonal behavior. At the conceptual level, we conclude that the replicated finding (within two empirical case studies) of a higher complexity than originally assumed by the classical symptom specificity hypothesis, suggests areas for potential hypothesis refinement.

At the methodological level, we conclude that single case research, in which extensive multiple method and multiple source data sets on one patient are analyzed, is required to grasp the complex, clinical interplay between symptoms and interpersonal dynamics, and to indicate on which points the classical hypothesis needs refining.

However, the suggested complexity needs to be further investigated. Future nomothetic research efforts should aim to test if the proposed refinement can be *statistically generalized* to broader populations of obsessional subjects. Further, endeavors to enhance confidence in the *clinical utility* of the symptom specificity hypothesis, by means of additional (series of) case studies, which more closely resemble everyday practice, are believed to be a necessary complement. Finally, to stimulate further improvements in the theory (e.g., Stiles, 2015), it would be valuable to contrast our findings to results from future longitudinal case studies as to whether (dis)similar patterns can be found in the underlying processes that are responsible for interpersonal and symptomatic alterations (see also Iwakabe and Gazzola, 2009).

## Ethics statement

Ethics Committee of Ghent University Hospital. Consent for data-collection, data-analysis and publication of individual case materials was provided by Ethics Committee of Ghent University Hospital. The specific dossier that was approved by the Ethics Committee can be given upon request.

## Author contributions

SC: Creation of the conception and design of the study; contribution to data-collection; main data-analyst; main interpretor of the data; main author of the manuscript. MD: Creation of the conception and design of the study; contribution to data-collection; main reviewer of the manuscript. KV: Contribution to data-collection; data-analyst; interpretor of the data. RM: Contribution to data-collection; data-analyst; reviewer of the manuscript. JW: Contribution to data-collection; data-analyst; reviewer of the manuscript. RI: Contribution to data-collection; data-analyst; reviewer of the manuscript. JF: Contribution to data-collection; reviewer of the manuscript.

### Conflict of interest statement

The authors declare that the research was conducted in the absence of any commercial or financial relationships that could be construed as a potential conflict of interest.
